# Perspective: large language models and antimicrobial resistance among migrants: an equity imperative

**DOI:** 10.1038/s41746-026-02742-y

**Published:** 2026-05-14

**Authors:** Ji Soo Choi, Daniel Pan, Anthony O’Hare, Catrin E. Moore, Fouad Fouad, Manish Pareek

**Affiliations:** 1 Development Centre for Population Health, University of Leicester, Leicester, UK; 2 Department of Respiratory Sciences, University of Leicester, Leicester, UK; 3 Leicester NIHR Biomedical Research Centre, Leicester, UK; 4 Department of Infectious Diseases and HIV Medicine, Leicester, UK; 5 Computing Science and Mathematics, Faculty of Natural Sciences, University of Stirling, Stirling, UK; 6 Centre for Neonatal and Paediatric Infection, Institute for Infection and Immunity, City St George’s, University of London, London, UK; 7 Liverpool School of Tropical Medicine, Liverpool, UK; 8 Centre for Ecology and Hydrology, Wallingford, UK; 9 Imperial College London, London, UK; 10 University of New Mexico, Albuquerque, NM USA; 11 NHS Cheshire and Merseyside, Liverpool, UK; 12 North West NHS England, Manchester, UK; 13 St Helens and Knowsley Teaching Hospitals NHS Trust, Prescot, UK; 14 University of Edinburgh, Edinburgh, UK; 15 University of Leeds, Leeds, UK; 16 University of Liverpool, Liverpool, UK; 17 Untap Health, London, UK

**Keywords:** Antimicrobials, Mathematics and computing

## Abstract

Despite progress in antimicrobial resistance (AMR) surveillance, migrants and ethnic minorities, who bear disproportionate AMR burdens, remain underrepresented in programmes. Digital health is common, but we found no interventions using large language models (LLMs) to reduce AMR in these communities. In three workshops, we identified priorities: culturally and linguistically inclusive design; context specific knowledge from community settings; and trust building via community health workers, with data protection and bias mitigation.

## Migrants remain under-recognised in AMR strategies and surveillance

Rapid advances have been made in antimicrobial resistance (AMR) surveillance, infection prevention and control and antimicrobial stewardship, yet those who shoulder a disproportionate risk of and thus share of the AMR burden—migrants, refugees and asylum-seekers—remain relatively invisible in global initiatives on reducing AMR^1^. For instance, migrant populations remain under-represented in World Health Organisation action plan on AMR, and Global Antimicrobial Resistance and Use Surveillance System (GLASS) does not adequately include data on migrant populations^2^. Over one-quarter of migrants in Europe are estimated to carry or be infected with drug-resistant pathogens, with greater prevalence seen in community settings than in hospitals (41% vs. 21%, respectively). Furthermore, there is little evidence of onward transmission to receiving populations; rather, migrants themselves face increased risks of exposure during transit and in receiving countries due to poor conditions and barriers to care^3^. When comparing migrants to non-migrants, the prevalence of AMR differs significantly: one cohort study in Denmark analysing antibiotic resistance patterns of *Escherichia coli* in over 14,000 urine samples found Ciprofloxacin resistance in 5.8% of migrants and 2.2% of cases non-migrants respectively, and a similar pattern was observed with Gentamicin (10.8% vs. 4.7%) and Cefuroxime (8.5% vs. 3.4%)^2,4^.

## Why AMR risk is elevated in migrant communities

**Fig. 1:**
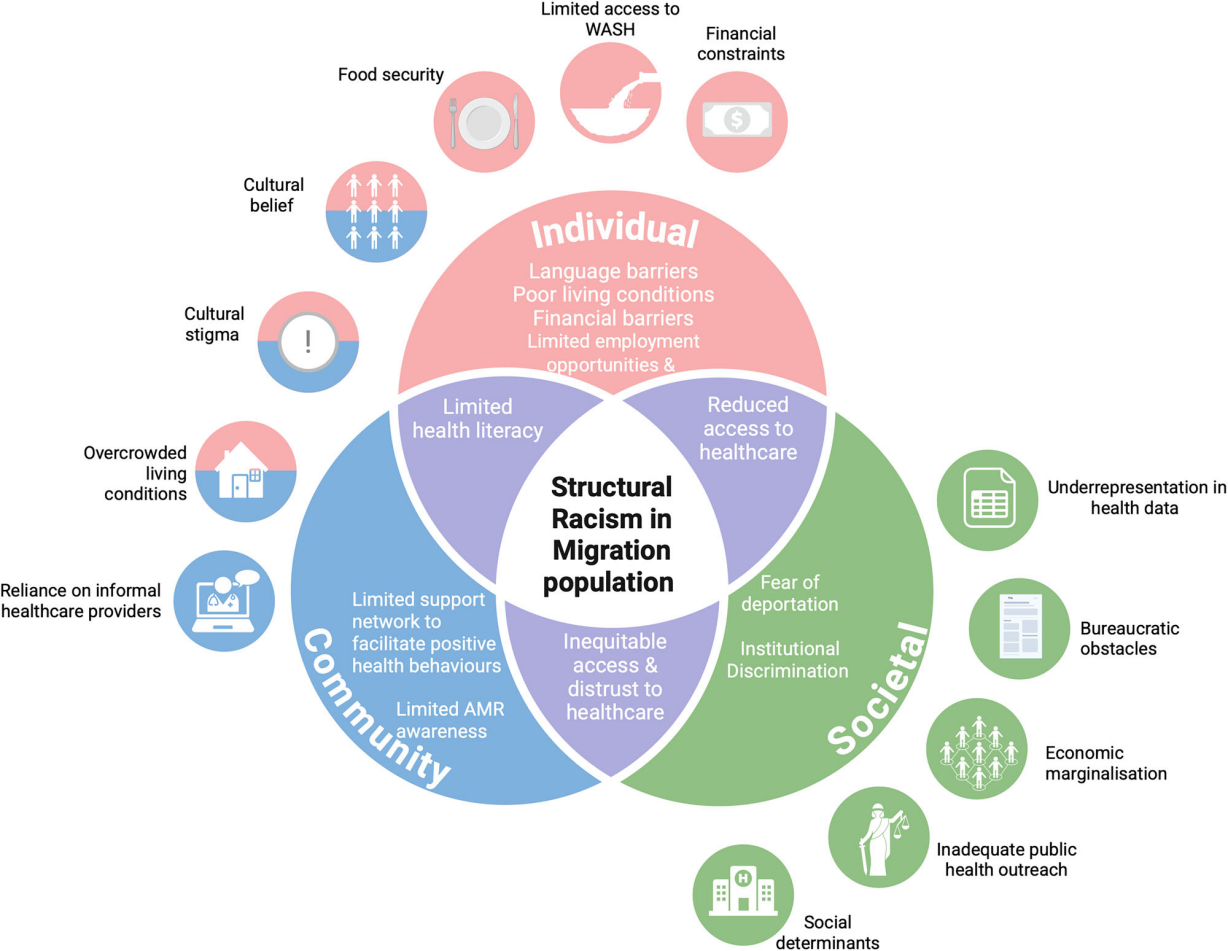
Drivers of antimicrobial resistance (AMR) in migrant populations. Interacting individual, community, and societal drivers of antimicrobial resistance in migrant population with structural racism as an underlying factor. Created in BioRender. Pan, D. (2026) https://BioRender.com/9l146ef.

Migrants are a heterogenous group. Forced migration broadly stems from natural or human-made disasters including climate change; conflict, violence, persecution, or other violations of human rights; and multi-dimensional insecurity (i.e., economic, political, social) in the country of origin. Increasing and unprecedented rates or forced migration globally have been mirrored by increasingly restrictive migration, health, and social policies in migrant-receiving countries^5–7^. The COVID-19 pandemic also contributed to more hostile attitudes towards migration and minority communities^8^, and resulted in significant delays in or access to essential immigration or social services, major socio-economic barriers and health insecurity for migrant populations, and key public health consequences including compromised access to basic water, sanitation, and hygiene (WASH) services, housing, vaccination, and healthcare^9^. For instance, the pandemic increased antibiotic prescribing via telehealth, greater use of prophylactic antibiotics, and widespread concern about secondary bacterial infections in COVID-19 patients. These trends were observed globally, including in countries of origin for many migrants, not only in high-income receiving countries. As a result, migrants may be more likely to seek antibiotics through informal or unregulated channels and may feel pressure to present with symptoms that increase the likelihood of receiving a prescription^10,11^. This inequity is further compounded by community and societal level barriers, such as exclusionary eligibility rules, language discordance, insecure legal status, and the legacy of discriminatory care. Together, these contribute to marginalised communities being outside formal healthcare and surveillance systems, which perpetuates AMR in migrant populations (Fig. 1)^12,13^.

## A clear research and implementation gap for LLMs

With growing barriers faced by the migrant population imposed by current policies, and as the effects of such marginalisation impact public health, the gap in attention to AMR in these communities demands immediate action through more inclusive and adaptive solutions. Digital health technologies have been widely implemented across health research and can be powerful approaches for utilising heterogeneous data and identifying patterns across diverse populations. Yet, the use of artificial intelligence to address AMR in marginalised groups remains insufficiently explored. Although LLMs have been used within the context of underserved communities to understand dynamics and public attitudes towards health policies and interventions for predicting outbreaks and improving patient communication^14^, their application and value is under-investigated and under-utilised in the intersection between AMR and migration^15^.

We performed a search of MEDLINE using Ovid, combining the terms “migrants”, “antimicrobial resistance”, and “large language models” from database inception until 3rd January 2026 and identified no relevant original research publications, highlighting a notable research gap (full search strategy in supplementary materials). To date, no interventions have been performed leveraging LLMs to design programmes that aim to reduce the burden of AMR among disadvantaged populations, representing a missed opportunity to address an urgent global health issue and advance equity and inclusivity. Many reasons could exist for why LLMs have not yet been applied to address the issue of AMR in at-risk populations. Most notably, there remains the danger of developing LLMs that are safe and accurately represent such a diverse and heterogeneous group of vulnerable cohorts. Furthermore, the use of LLM involves inferring information from data; within certain migrant or underserved populations, the performance and generalisability of algorithms are constrained by the adequacy and representativeness of such limited data. As such, making predictions beyond the observed data can be problematic.

## What LLMs already do for AMR and what is missing for migrants

Artificial intelligence is fundamental for addressing challenges related to AMR^16^. LLMs have been developed for drug discovery and development of new antibiotics (e.g., the use of neural networks to predict whether a new compound could potentially inhibit microbial growth; such as the discovery of halicin against *Escherichia coli* or abaucin against *Acinetobacter baumanii*)^17^. Clinical decision support systems leverage the use of LLM to diagnose and treat common bacterial infections. One recent randomised multimethod study found that artificial intelligence driven clinical decision support systems were positively received by clinicians and has the potential to support antimicrobial prescribing, with the greatest influence on clinicians when it recommended not switching from intravenous to oral treatment, which can implications on the development of AMR (logistic regression odds ratio of not switching: 0.13, 95$ CI: 0.03–0.50, *p* = 0.003)^18^. Within mathematical modelling, the dynamics of susceptible and resistant bacteria between and within hosts can be integrated into mathematical modelling frameworks to identify short-term and long-term effects of antimicrobial therapy at individual and population levels. Determining and weighing the overall impact of AMR transmission on individuals, populations, and healthcare systems can be challenging and could be greatly improved with the use of LLMs^19^. And yet, there remains absolutely not use of LLMs to address AMR within those who are arguably the most disproportionately affected of all patient cohorts.

Three key factors must be taken into account when developing LLMS to design interventions that could address AMR within migrant and ethnic minority groups^20^.

### Cultural and linguistic diversity

First, embracing cultural and linguistic diversity is essential. Migrant communities communicate across a wide range of languages, dialects, and cultural expressions when describing symptoms and medications, with unique health beliefs and illness models^21^. Therefore, LLMs must be trained on multilingual and culturally grounded datasets such as social media or unpublished media to account for heterogenous use of social platforms and capture “lay” language and terminology. Without proper adaptation, a generic model may not fully capture the diversity of migrants and their specific needs, leading to ineffective strategies^22^. Fine-tuning with language data from migrant communities—and incorporating input from local community health workers, translated clinical interactions, and qualitative data—can help models learn local expressions for illnesses and antibiotics to build the necessary cultural sensitivity.

### Socio-behavioural context

Second, integrating socio-behavioural context is critical to effectiveness. Adaptive behaviours such as saving antibiotics for later use, sharing medications, and seeking informal care reflect structural barriers such as mistrust, difficulties in accessing healthcare, or financial challenges, rather than individual preference^3,23^. Context-specific knowledge bases, drawn from environments such as community organisations, refugee camps and transit hubs, can equip LLMs to better model the real-world decision-making processes of migrants, enabling more precise, culturally attuned support and interventions. This can be implemented with techniques like retrieval-augmented generation, which can supply the model with up-to-date, community-specific information from interactions between migrants and healthcare workers^22^. Additionally, data from migrants’ own narratives, such as anonymised social media posts, survey responses about healthcare experiences, and qualitative research, could be fed into the model to enrich its understanding of their perspectives. By training on diverse inputs, an LLM can act as a virtual mirror, reflecting the individual, community, and structural barriers that migrant patients encounter, providing solutions and possible interventions.

### Trust as a core design principle

Building trust must be a central design principle. For migrant communities, whose experiences with healthcare systems are shaped by discrimination, language barriers, institutional neglect, and explicit ‘hostile environment’ policies that may implement enforcement strategies such as data sharing, trust is fragile, and it must be rebuilt through sustained commitment to equity and inclusion^23^. Migrant communities are diverse and represent marginalised populations across the world. There are currently over 300 million migrants worldwide, and clear differences exist between those who declare, or do not declare refugee status^24^. The origin of migrants to a single new host country also varies across time; depending on where poverty and/or global conflict exist in the world. For example, the characteristics of Somali refugees resettling in Western European countries in the 1990s will be very different from Syrian migrants that migrated to Germany^25,26^. Furthermore, the needs of migrant populations can be completely different depending on the country they migrate to; some migrants can only migrate to nearby countries, since they are too poor, sick or disconnected to migrate further; many will remain in camps or die during transit. Specifically for AMR, many low-middle-income countries have pharmacies that sell antibiotics directly to patients without medical supervision for a range of conditions, or have counterfeit medications which have suboptimal doses of the actual drug^27^. Migrants which spend time in refugee camps may also have suboptimal access to emergency medical and as well as expired or illegitimate antibiotics^28^.

LLMs have the potential to bridge some of these historical gaps by providing culturally respectful communication, validating individual and community narratives, and responding transparently when uncertainty or risks arise. To enable this, LLMs should be trained on multilingual, culturally specific data and supported by oversight from community health workers to ensure cultural fidelity, ethical responsiveness, and timely human intervention when needed. Beyond accurate translation or information retrieval, models must actively reflect and validate migrant experiences, addressing individual, community, and organisational barriers to healthcare access—ultimately strengthening trust and engagement over time. It is imperative that the use of migrant data and its implementation through technologies like artificial intelligence are rigorously protected and used to benefit migrant and public health, not as a mechanism for immigration enforcement or the further marginalisation of these communities.

## Ethics, governance, and anti-racist data protection

Importantly, the deployment of LLMs must be based on good-quality data collected from migrant populations, which must be grounded in an anti-racist framework, rather than simply aiming for cultural competence. More specifically, LLMs must be developed in a way that aims to improve access to timely healthcare. Models should be designed to actively dismantle biases and promote equity in how migrant health data are collected, interpreted, and acted upon. The LLM must be able to interpret the inputs from migrants accurately, to minimise medical errors in responses, or misunderstanding of responses, which could perpetuate mistrust. The ethical risks of an inaccurate LLM are substantial: algorithmic bias, data misuse, surveillance creep, and the temptation to substitute chatbots for structural reform. Developing a LLM that focuses on improving trust and access to care has the potential for application in migrants beyond the field of AMR.

Along with ethical challenges, the limited availability of electronic data on diverse experiences and dialogues used by minoritised populations raises concerns about inherent biases and prejudices present in training data. Such bias, stemming from insufficient data, risks causing LLMs to generate skewed outputs and reinforce cultural or racial stereotypes, potentially exacerbating existing inequalities. To address this, the Synthetic Minority Oversampling Technique (SMOTE) can be employed to augment existing data to generate more examples of target culture through prompt engineering^29^. This approach enriches the data used to train LLMs, promoting fairness and equity while minimising the risk of misclassification. Over time, LLMs can acquire real-time data from simulations and feedback from qualitative research, which can eventually replace data generated by SMOTE. However, it is important to consider that LLMs often mirror historical biases present in human language, and the use of SMOTE risks amplifying these biases within automated systems. Therefore, a selective and careful approach is required to avoid mischaracterisation or exclusion of certain groups.

Leveraging the use of LLM aligns with the United Nations Global Compact for Migration, whose primary objective is to “reduce the risks and vulnerabilities migrants face at different stages of migration” through 23 supportive strategies. Specifically, this LLM corresponds to one of its 23 action plans, which seeks to “Strengthen certainty and predictability in migration procedures for appropriate screening, assessment and referral”^30^. Initially, the LLM could serve migrants from low- to middle-income countries arriving in Europe, given the recent rise in migration to the EU^30^. However, past experience with digital health and border databases—from EURODAC, the first biometric database containing fingerprints of all asylum applicants of Europe, to the former UK policy of using NHS patient records in immigration enforcement—shows how tools designed for protection can be repurposed for surveillance, with detrimental consequences for these communities^31,32^.

## From principles to implementation: co-production and safeguards

Generating LLMs representative of migrant experiences requires co‑creation and co‑production: migrants must be at heart of the data, the algorithms and the applications that emerge. Without such migrant-led governance, any promise of safer, fairer decision-making risks becoming yet another lever for authorities to track and penalise the very people the system is meant to serve. This requires a mixed-methods, pan-disciplinary research approach, with continual input from migrant populations. This will ensure a bidirectional process, from building a diverse, intergenerational knowledge base to inform model training, to repeated interrogations of early model prototypes, working directly with developers to continually refine the LLM^14^. This reinforcement learning with human feedback will not only improve technical performance but also nurture “perceived identity of belonging”; a subjective sense that migrants are accepted, included and are core to the project, thereby rebuilding trust in a healthcare landscape where trust is often fragile^33^.

In summary, structural and inequities, alongside individual and community-level barriers, continue to drive the risk and burden of antimicrobial resistance among migrant populations. By incorporating diverse cultural, behavioural, and lived experiences into simulations, communication strategies, and surveillance models, LLMs offer a novel opportunity to address these systemic inequities. With intentional, equity-driven design, LLMs have the potential to transform migrants from marginalised groups into empowered partners in AMR surveillance and stewardship, and produce insights that inform adaptive actions of communities and local and national public health and social welfare actors. Importantly, when developing LLMs to address AMR in migrants, we must recognise that they are an incredibly underserved, yet heterogenous population. It is not possible to develop one LLM to address AMR in all migrants; rather, we must apply the above principles with bespoke data in accordance with the time, location and characteristics of the specific migrant or underserved groups in mind. Artificial intelligence should be positioned as a catalyst for advancing migrant health equity and strengthening global AMR responses.

## Supplementary information

Supplementary materials

## Data Availability

No datasets were generated or analysed during the current study.
