# Supplementary material for: Perspective: large language models and antimicrobial resistance among migrants: an equity imperative
Source: NPJ Digit Med. 2026 May 14;9:606. doi: 10.1038/s41746-026-02742-y (PMC13448168; doi:10.1038/s41746-026-02742-y)
Supplement: Supplementary file 1 [file 41746_2026_2742_MOESM1_ESM.pdf]

Supplementary materials: Search strategy of MEDLINE using OVID. Yellow highlights are the combined searches that are used

Ovid MEDLINE(R) ALL <1946 to Jan 03, 2026>

1 "transients and migrants"/

2 migrant*.mp.

3 expatriate.mp.

4 departee.mp.

5 asylum.mp.

6 foreign-born.mp.

7 foreign born.mp.

8 foreign worker.mp.

9 foreign student.mp.

10 international student.mp.

11 sex traffick*.mp.

12 ((wom?n or child* or man or men) adj2 traffick*).mp.

13 or/1-12

14 Drug Resistance, Bacterial/

15 ((antibiotic* or anti-bacterial or anti-microbial) adj3 resistan*).mp. [mp=title, book title, abstract, original title, name of substance word, subject heading word, floating sub-heading word, keyword heading word, organism supplementary concept word, protocol supplementary concept word, rare disease supplementary concept word, unique identifier, synonyms, population supplementary concept word, anatomy supplementary concept word]

16 Drug Resistance, Microbial/

17 14 or 15 or 16

18 Large Language Models/

19 (large language model* or machine learning).mp.

20 Artificial Intelligence/

21 artificial intelligence.mp.

22 chat gpt.mp. or Generative Artificial Intelligence/

23 generative ai.mp.

24 or/18-23

25 13 and 17 and 24

26 (ethnicity or ethnic).mp. [mp=title, book title, abstract, original title, name of substance word, subject heading word, floating sub-heading word, keyword heading word, organism supplementary concept word, protocol supplementary concept word, rare disease supplementary concept word, unique identifier, synonyms, population supplementary concept word, anatomy supplementary concept word]

27 Minority Groups/

28 continental population groups/

29 hispanic americans/

30 african continental ancestry group/

31 American Native Continental Ancestry Group/

32 Asian Continental Ancestry Group/

33 European Continental Ancestry Group/

34 Oceanic Ancestry Group/

35 African Americans/

36 Arabs/

37 Asian Americans/

38 (multi?cultural or multi cultural or cross?cultural or cross cultural or trans?cultural or transcultural).mp. [mp=title, book title, abstract, original title, name of substance word, subject heading word, floating sub-heading word, keyword heading word, organism supplementary concept word, protocol supplementary concept word, rare disease supplementary concept word, unique identifier, synonyms, population supplementary concept word, anatomy supplementary concept word]

39 (BAME or minority or minorities).mp. [mp=title, book title, abstract, original title, name of substance word, subject heading word, floating sub-heading word, keyword heading word, organism supplementary concept word, protocol supplementary concept word, rare disease supplementary concept word, unique identifier, synonyms, population supplementary concept word, anatomy supplementary concept word]

40 exp Population Groups/

41 "transients and migrants"/

42 migrant*.ti,ab,kw,kf.

43 expatriate*.ti,ab,kw,kf.

44 asylum.ti,ab,kw,kf.

45 foreign-born.ti,ab,kw,kf.

46 indigenous.ti,ab,kw,kf. or Indigenous Peoples/

47 Ethnic Groups/

48 refugee*.ti,ab,kw,kf. or Refugees/

49 aboriginal*.ti,ab,kw,kf.

50 "country of birth".ti,ab,kw,kf.

51 ((Minorit* or Mixed) adj3 (ethnic* or race)).ti,ab,kw,kf.

52 (Arab* or Africa* or Afro* or Asian or Bangladesh* or Black or Caribbean or Chinese or India* or Irish or (Mixed adj other) or Multi-rac* or Pakistan* or Roma or traveller* or traveler* or Gyps* or Gips*).ti,ab,kw,kf.

53 (multicultural or "multi cultural" or multiethnic or "multi ethnic" or multiracial or "multi racial" or crosscultural or "cross cultural" or "trans cultural" or transcultural or biracial).ti,ab,kw,kf. 41781

54 immigrant*.ti,ab,kw,kf.

55 (Ethnic* or racial or race or racis* or "people of color" or "people of colour" or POC or "BME" or "BAME").ti,ab,kw,kf.

56 or/26-55

57 17 and 24 and 56

58 Patient Education as Topic/ or health literacy.mp. or Health Education/ or Health Knowledge, Attitudes, Practice/ or Health Literacy/ 288328

59 (attitude* or perception* or understanding*).tw. 1865003

60 58 or 59 2075498

61 17 and 24 and 56 and 60
